# Editorial: Non-coding RNA in immunotherapies and immune regulation

**DOI:** 10.3389/fimmu.2022.1094643

**Published:** 2022-11-29

**Authors:** Bertrand Kaeffer, Chen Chen, Antoine Louveau

**Affiliations:** ^1^ Département Alimentation Humaine, Nantes Université, Institut national de recherche pour l’agriculture, l’alimentation et l’environnement (INRAE), Unité Mixte de Recherche (UMR) 1280, Nantes, France; ^2^ Tongji Hospital, Tongji Medical College, Huazhong University of Science and Technology, Wuhan, China; ^3^ Lerner Research Institute, Cleveland Clinic, Cleveland, OH, United States

**Keywords:** ncRNA, circular RNA, ce-RNA, diabetes, extracellular vesicles, inflammation, sepsis, Covid-19

## Introduction

The immune system is a key player in mammal homeostasis, where non-coding RNAs are involved in the differentiation and regulation of immune cells as well as in epigenetics mechanisms. Non-coding RNAs are divided into short-chain noncoding RNAs with a length of 18-200 nt and long-chain noncoding RNAs (lncRNAs) with a length >200 nt (1). Short-chain noncoding RNAs mainly include microRNAs (miRNAs), piwi-interacting RNAs, small nucleolar RNAs, small nuclear RNAs and repeat RNAs. The description of lncRNAs is in expansion, and some have been linked to epigenetic mechanisms. microRNAs bind to sequences with partial complementarity on target RNA transcripts, called microRNA recognition elements (MREs). Coding and non-coding RNA targets can cross-talk through their ability to compete for microRNA binding. Through MREs, transcripts can actively communicate to each other to regulate their respective expression levels with consequences on a large proportion of the transcriptome (2).

**Figure 1:**
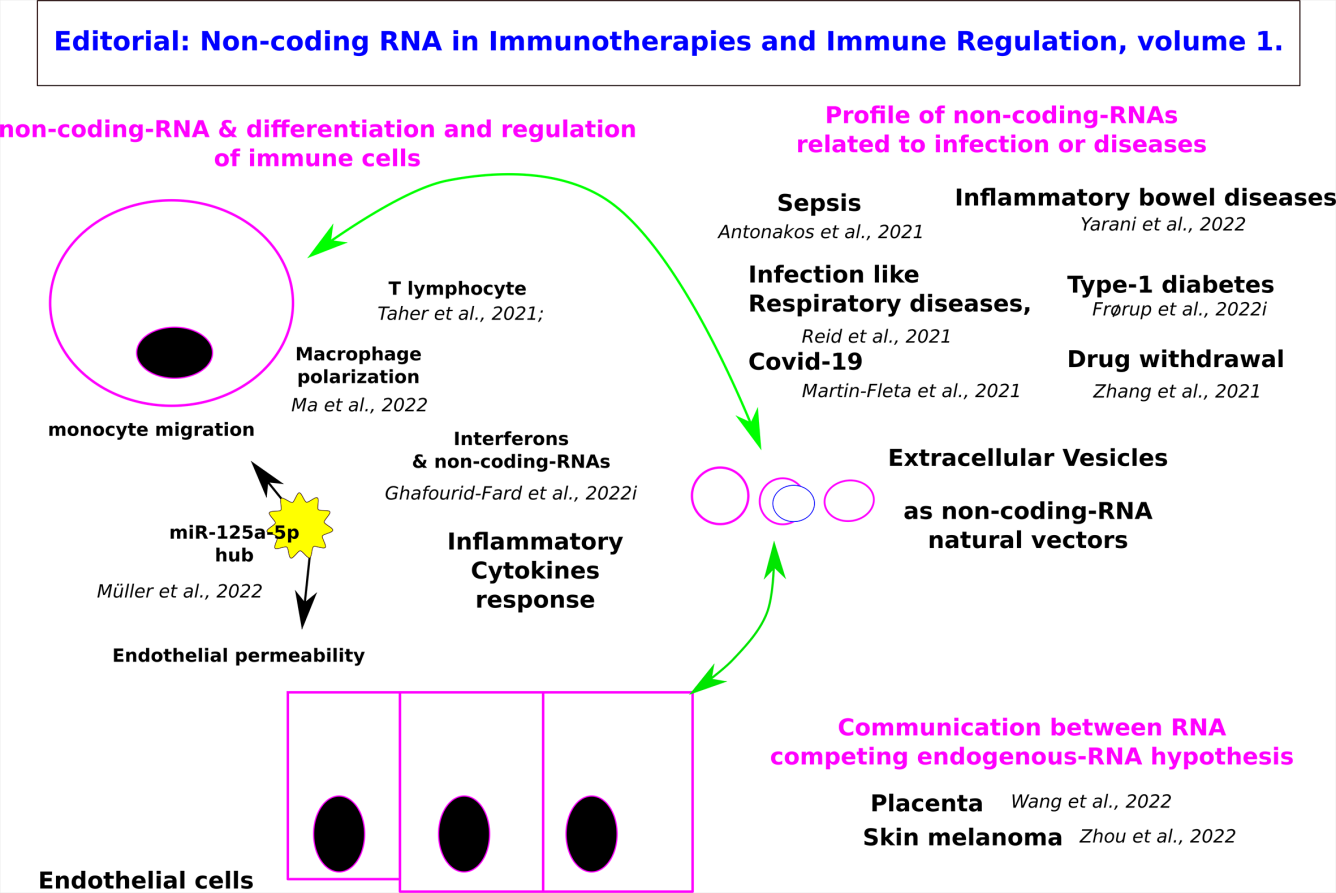
Graphical abstract of the Editorial: Non-coding RNA in Immunotherapies and Immune Regulation.

This e-book through the valuable contributions of the authors is paving the way for the development of RNA-based therapeutics in cancer, metabolic and infectious diseases (**Figure 1**).

Müller et al. propose that miR-125a-5p acts as a hub for endothelial barrier permeability and monocyte migration. Inflammatory stimulation of endothelial cells induces miR-125a-5p expression, which consecutively inhibits a regulatory network consisting of the two adhesion molecules VE-Cadherin and Claudin-5, two regulatory tyrosine phosphatases (PTPN1, PPP1CA) and the transcription factor ETS1. During macrophage polarization, miR-125a-5p also reduces the expression of M1 phenotype induced by LPS and promotes the expression of M2 induced by IL-4 by targeting KLF13 to regulate the phagocytosis and bactericidal activity of macrophages (Ma et al.). Interferon-gamma (IFNG) is one of the most important mediators of immunity and inflammation and plays a key role in macrophage activation, inflammation, host defense against intracellular pathogens, Th1 cell response, and tumor surveillance. T-lymphocytes play a major role in adaptive immunity and current immune checkpoint inhibitor-based cancer treatments (Taheri et al.; Antonakos et al.; Ghafouri-Fard et al.). IFNG-antisense-1 (IFNG-AS1) is a lncRNA that participates in the regulation of IFN responses. Located downstream of the IFNG locus, its expression correlates strongly with the expression of IFNG. CD4+ and CD8+ T cells as well as NK cells express this lncRNA. The impact of lncRNAs on interferon signaling has also been assessed in the context of diabetes mellitus. Lnc10 contains a type I diabetes-associated single nucleotide polymorphism. This lncRNA can regulate the expression of the IRF7-driven inflammatory network regulating gene Ebi2 in immune cells IRF (Interferon Regulatory Factor). IFNG-AS1, lnc-ITSN1-2, lncRNA-CD160, NEAT1, MEG3, GAS5, NKILA, lnc-EGFR, and PVT1 are among lncRNAs that efficiently influence the function of T cells. Moreover, the effects of a number of circular RNAs, namely circ_0001806, hsa_circ_0045272, hsa_circ_0012919, hsa_circ_0005519 and circHIPK3 are ascertained in the modulation of T-cell apoptosis, differentiation, and secretion of cytokines (Taheri et al.). The circular RNAs, novel non-coding RNAs produced by reverse splicing of mRNA precursors, are currently explored for therapeutic applications (3).

The apoptosis, proliferation, or migration of endothelial cells, as well as the inflammatory status, are regulated by endogenous or microvesicle-derived miRNAs. For example, miR-155 increases in pulmonary endothelial cells of sepsis mice targeting the tight junction protein Claudin-1, and induces capillary leakage during infection (Antonakos et al.) but this miRNA is also involved in IFNG regulation (Ghafouri-Fard et al.). Likewise, platelet microparticles containing miR-223 reduce intercellular adhesion molecule 1 expression and binding to peripheral blood mononuclear cells by endothelial cells, providing a possible protective role against excessive sepsis-induced vascular inflammation. miR-223-3p regulates peroxisome proliferator-activated receptor-γ mediated M2 macrophage activation. Overall, many miRNAs are associated with the polarization/activity of M1 macrophages like miR-155-5p, and M2 macrophages like miR-125a-5p, and miR-223. Yarani et al. show that miR-223-3p, miR-16-5p, and miR-24-3p are upregulated in both mucosa and blood of Ulcerative Colitis patients. In Crohn Disease, miR-223-3p and miR-21-5p are upregulated in both mucosa and blood of patients. miR-223-3p is involved in the activation of granulocytes and is overexpressed in naive CD4+ T- lymphocytes. miR-223-3p abundance in macrophages can change macrophage activation and modulate the response to stimuli *via* effects on the TLR4/FBXW7 axis. miR-223-3p is now considered a biomarker in Intestinal Bowel Disease that seems to be conserved between different species.

Coronavirus Disease 2019 (COVID-19) pneumonia is a life-threatening infectious disease, especially for elderly patients with multiple comorbidities (4). Multivariate analysis displayed a combination of 4 miRNAs (miR-106b-5p, miR-221-3p, miR-25-3p and miR-30a-5p) that significantly discriminated between both pathologies (Martinez-Fleta et al.). miR-335-5p is significantly downregulated in COVID-19. EGFR, a known target of miR-27b-3p, miR-146a-5p, miR-16-5p, miR-335-5p and miR-30a-5p, is found at higher levels in patients with COVID-19. Increased levels of the chemokine CXCL12 in Community-Acquired Pneumonia vs. COVID-19 patients are found. CXCL12, which is the CXCR4 ligand, is necessary for effective hematopoiesis, T cell, and memory B cell homing to the lymph nodes or monocyte recruitment. Inhibition of this axis is used by several viruses in order to increase their proliferation by reducing the number of circulating immune cells. Reid et al. have explored the potential role of Extracellular vesicles (EVs) as circulating inflammatory mediators which could propagate systemic inflammation and multimorbidity in response to shared risk factors including aging, inhaled noxious stimuli and respiratory infections. The intercellular transfer of EV miRNA has therefore been implicated in mediating a range of pathophysiological processes including in the development of COPD. Profiling miRNAs through Evs in the plasma of type-1 diabetic mothers has been done with hsa-miR-146a-5p, hsa-miR-26a-5p, hsa-miR-24-3p, and hsa-miR-30d-5p (Frørup et al.). The sorting mechanisms for MicroRNAs into Extracellular Vesicles and their Associated Diseases have been recently reviewed (5). Likewise, the dynamics of lncRNAs and mRNAs transported by EVs in Heroin addicts during acute and protracted withdrawal have been explored as a first step toward improved therapy (Zhang et al.).

In cancer, the potential diagnostic and prognostic markers related to Skin melanoma of the OIP5-AS1-hsa-miR-186-5p/hsa-miR-616-3p/hsa-miR-135a-5p/hsa-miR-23b-3p/hsa-miR-374b-5p-PTPRC/IL7R/CD69andMALAT1-hsa-miR-135a-5p/hsa-miR-23b-3p/hsa-miR-374b-5p-IL7R/CD69 ceRNA networks have been proposed (Zhou et al.). On placenta of mothers suffering from Pregnancy-related intrahepatic cholestasis, immune-related genes KLRD1, BRAF, and NFATC4 might have a potential ceRNA mechanism by individual lncRNA sponging miR372-3p, miR-371a-3p, miR-7851-3p, and miR-449a to control downstream the level of TNF-a, IFN-g, and IL-10 (Wang et al.).

In conclusion, RNAs are able to interact with DNAs, RNAs, and proteins bringing up biochemical networks within a single cell or connecting multiple cell types. Extensive validation studies will be needed to consider the side effects of RNA-based drugs (6), as well as detailed descriptions of the Competing Endogenous RNA network (ce-RNA) underpinning immune cell regulation. Designing new therapeutics from complex biological regulation driven by RNA machinery will benefit from the reappraisal of selective pressure on the machinery at the level of genome regulation or to ward off the cell from transposable elements or viruses (7). Likewise, designing new mathematical models of immunity (Bocharov et al.) integrating non-coding RNAs will be of utmost importance.

## Author contributions

All authors listed have made a substantial, direct, and intellectual contribution to the work and approved it for publication.

## Acknowledgments

We wish to thank all authors who contributed to this Research Topic, as well as all reviewers and editors outside the board, for their dynamic and positive contributions.

## Conflict of interest

The authors declare that the research was conducted in the absence of any commercial or financial relationships that could be construed as a potential conflict of interest.

## Publisher’s note

All claims expressed in this article are solely those of the authors and do not necessarily represent those of their affiliated organizations, or those of the publisher, the editors and the reviewers. Any product that may be evaluated in this article, or claim that may be made by its manufacturer, is not guaranteed or endorsed by the publisher.
